# Resting-State Functional Connectivity Predicts Cognitive Impairment Related to Alzheimer's Disease

**DOI:** 10.3389/fnagi.2018.00094

**Published:** 2018-04-13

**Authors:** Qi Lin, Monica D. Rosenberg, Kwangsun Yoo, Tiffany W. Hsu, Thomas P. O'Connell, Marvin M. Chun

**Affiliations:** ^1^Department of Psychology, Yale University, New Haven, CT, United States; ^2^Interdepartmental Neuroscience Program, Yale School of Medicine, New Haven, CT, United States; ^3^Department of Neuroscience, Yale School of Medicine, New Haven, CT, United States

**Keywords:** aging, Alzheimer's disease, mild cognitive impairment, functional connectivity, resting state

## Abstract

Resting-state functional connectivity (rs-FC) is a promising neuromarker for cognitive decline in aging population, based on its ability to reveal functional differences associated with cognitive impairment across individuals, and because rs-fMRI may be less taxing for participants than task-based fMRI or neuropsychological tests. Here, we employ an approach that uses rs-FC to predict the Alzheimer's Disease Assessment Scale (11 items; ADAS11) scores, which measure overall cognitive functioning, in novel individuals. We applied this technique, connectome-based predictive modeling, to a heterogeneous sample of 59 subjects from the Alzheimer's Disease Neuroimaging Initiative, including normal aging, mild cognitive impairment, and AD subjects. First, we built linear regression models to predict ADAS11 scores from rs-FC measured with Pearson's *r* correlation. The positive network model tested with leave-one-out cross validation (LOOCV) significantly predicted individual differences in cognitive function from rs-FC. In a second analysis, we considered other functional connectivity features, accordance and discordance, which disentangle the correlation and anticorrelation components of activity timecourses between brain areas. Using partial least square regression and LOOCV, we again built models to successfully predict ADAS11 scores in novel individuals. Our study provides promising evidence that rs-FC can reveal cognitive impairment in an aging population, although more development is needed for clinical application.

## Introduction

Cognitive decline occurs in both normal aging and neurodegenerative disorders (Hedden and Gabrieli, 2004; Jagust, 2013) and has a profound impact on individuals' quality of life as well as life satisfaction (St John and Montgomery, 2010; Abrahamson et al., 2012). Elucidating the neural processes underlying such decline is of critical importance to developing strategies for healthy aging and treatments for neurodegenerative disease. However, the brain aging process accompanying such cognitive decline in normal aging is characterized by a tremendous level of heterogeneity, with various extents of dysfunction in multiple brain systems, most notably the default-mode network (DMN), which is critical for memory and the frontoparietal network, which is critical for executive functioning (Ferreira and Busatto, 2013; Jagust, 2013). Critically such brain systems are also subject to influences by neurodegenerative disorders, such as Alzheimer's Disease (AD) and mild cognitive impairment (MCI) (Buckner, 2004; Badhwar et al., 2016). How can we characterize the different levels of cognitive decline using a neural marker despite such heterogeneity?

Recent efforts have been made to develop and validate aging- and AD-related neural markers that can be incorporated into clinical practice. Resting-state functional connectivity (rs-FC) measured with functional magnetic resonance imaging (fMRI) is a promising neuromarker for characterizing cognitive decline because it can reveal features of intrinsic functional brain organization relevant to cognitive abilities and disease status (Gabrieli et al., 2015; Woo et al., 2017). Compared to traditional diagnostic tools such as, neuropsychological tests, rs-fMRI may be less demanding on the participant. In addition, rs-fMRI does not involve the presentation of stimuli and thus is easier to standardize and share across different study sites.

Functional connectivity, the statistical interdependence between the blood oxygenation level dependent (BOLD) contrast signal time-courses of a pair of brain regions (Friston et al., 1994), has been used to assessed the intrinsic fluctuations of brain activity at rest (Biswal et al., 1995; Raichle et al., 2001). Functional alterations in resting-state networks have been implicated in AD and mild cognitive impairment (MCI) (Sheline and Raichle, 2013; Dennis and Thompson, 2014). However, most work on this topic focuses on a priori defined regions or networks such as the DMN and frontoparietal network (e.g., (Greicius et al., 2004; Sorg et al., 2007; Wang et al., 2007; Bai et al., 2009; Qi et al., 2010; Agosta et al., 2012; Damoiseaux et al., 2012; Vemuri et al., 2012). In addition, these studies typically investigate group differences among healthy control, MCI and AD participants and thus provide limited information about the cognitive impairment of individual subjects.

Recent advancements in rs-FC analyses demonstrate the exciting possibility of predicting an individual's cognitive abilities (e.g., sustained attention and fluid intelligence) from whole-brain FC (Finn et al., 2015; Rosenberg et al., 2016; Shen et al., 2017). Using Pearson's *r* as the measure of FC, Rosenberg et al. (2016) used connectome-based predictive modeling (CPM) to identify two functional networks related to sustained attention task performance: a high-attention network of functional connections positively correlated with performance on a sustained attention task across individuals, and a low-attention network of functional connections negatively correlated with task performance. Employing internal (leave-one-subject-out) and external (cross-dataset) validation procedures, they built predictive models based on the strength of these networks and showed that such models not only predicted task performance in novel individuals from both task-based and rs-fMRI, but also generalized to predict attention-deficit/hyperactivity disorder (ADHD) symptom severity in an independent group of children and adolescents from rs-fMRI (Rosenberg et al., 2016, 2017).

In another framework, Meskaldji et al. (2016) introduced two different measures of FC, accordance and discordance. Unlike Pearson's *r*, which is a summary measure of correlation between two regions, accordance and discordance respectively capture the correlation component and the anti-correlation component between the time-courses of two regions. Using partial least square regression (PLSR) and internal validation procedure, the authors demonstrated that accordance and discordance significantly predicted long-term memory scores in novel individuals in a group of MCI participants.

These results suggest that an individual's functional connectome—his or her unique pattern of whole-brain FC—contains important behavioral and clinical information. Since Alzheimer's disease and, more generally, the neurodegenerative process, evolve on a system level (Eidelberg and Martin, 2013), the whole-brain functional connectome should be particularly suitable for developing a neuromarker for clinically relevant cognitive decline in aging. Employing the two frameworks above (Pearson'r with CPM and accordance/discordance with PLSR), the current study sought to predict cognitive impairment related to Alzheimer's disease on an individual level in a heterogeneous sample including healthy control, MCI and AD participants tested at multiple study sites across the US. We additionally assessed and compared the performance of these different predictive models.

## Methods

### Participants

Data analyzed here were obtained from the Alzheimer's Disease Neuroimaging Initiative (ADNI) database (Weiner et al., 2010). ADNI was launched in 2003 as a public-private partnership with the primary goal of testing whether serial magnetic resonance imaging (MRI), positron emission tomography (PET), other biological markers, and clinical and neuropsychological assessment can be combined to measure the progression of MCI and early AD. ADNI consists of three phases: ADNI-1, ADNI-Grand Opportunity (ADNI-GO), and ADNI-2. For up-to-date information, see http://adni.loni.usc.edu/.

Because the ADNI-1 protocol did not include fMRI, all data in the current study were collected as part of ADNI-GO and ADNI-2. In this sample, 164 participants have at least one 7-min resting state fMRI scan, a corresponding magnetization prepared rapid gradient echo sequence (MPRAGE) scan, demographic information, and behavioral assessments available. Only data from the first visit with available 7-min fMRI scan were used in the current study. 103 participants were excluded due to excessive head motion during the resting state fMRI scan (defined a priori as >2 mm translation, >3° rotation, or >0.15 mm mean frame-to-frame displacement). An additional 2 participants were excluded because voxel size (2.5 × 2.5 × 2.5 mm) different from the standard ADNI fMRI voxel size (3.3 × 3.3 × 3.3 mm). The final sample included 59 participants (mean age = 72.53, range: 56–89; 31 females). The sample spanned a wide range of baseline diagnoses: (1) cognitively normal (CN), 14 participants; (2) significant memory concern (SMC), 5 participants; (3) early MCI, 14 participants; (4) late MCI, 15 participants; (5) AD, 11 participants.

### Alzheimer's disease assessment scale—cognitive subscales (ADAS-Cog)

We used the ADAS-Cog (11-item) score as the target variable for prediction. ADAS-Cog is a widely-used measure of cognitive performance in Alzheimer's disease trials. It measures impairments across several cognitive domains that are considered to be affected early and characteristically in Alzheimer's disease (Rosen et al., 1984). The 11-item version of ADAS-Cog (referred to as ADAS11) consists of the following items: word recall, commands, construction, naming, ideation praxis, orientation, word recognition, recall instructions, spoken language, word finding difficulty and comprehension. Higher ADAS11 scores indicate more severe cognitive impairment.

In the current sample, ADAS11 scores were not significantly correlated with age (Pearson's *r* = 0.04, *p* = 0.76) or average frame-to-frame displacement (Pearson's *r* = 0.13, *p* = 0.34). There is no difference in ADAS11 scores between male and female participants [*t*_(57)_ = −0.30, *p* = 0.76]^1^. ADAS11 scores were negatively correlated with years of education (Pearson's *r* = 0.25, *p* = 0.05) and therefore we controlled for the effects of years of education in our prediction (see section Education-Control Methods).

### Image acquisition

All imaging data were acquired on a 3-T Philips scanner at rigorously validated sites with a standardized protocol (Jack et al., 2010). Scan sessions included: localizer, sagittal MPRAGE, resting state fMRI, and axial PD and/or T2-weighted fast spin echo sequence. The ADNI MRI core optimized the acquisition parameters of these sequences for each model of scanner included in the study. All scanner sites were required to pass a strict scanner validation test before testing ADNI participants.

### Image preprocessing

Imaging data were preprocessed using AFNI (Cox, 1996). The first 3 frames were excluded. Data were despiked, slice-time and motion corrected. Mean signal from CSF in ventricles and mean global signal were regressed out from the data. A 24-parameter motion model, including six motion parameters, six temporal derivatives, and their squares, were also regressed from the data. The data were then detrended (quadratic trends) and band-pass filtered ([0.01 0.1 Hz]), to limit the analysis at the resting-state frequency range.

### Brain parcellation

Network nodes were defined using the Shen 268-node functional brain atlas, which includes the cortex, subcortex, and the cerebellum (Shen et al., 2013). Using the 3dWarp and 3dQwarp functions in AFNI (Cox, 1996), the atlas was warped from MNI space into single-subject space via concatenation of a linear and nonlinear registration between the functional images, anatomical scan, and the MNI brain. The two transformations were calculated independently and combined into a single transformation. After obtaining a subject-specific atlas, for every node, we calculated a mean time course by averaging the time courses of all of its constituent voxels using the roimeans function in BioImage Suite (Joshi et al., 2011) and obtained 268 mean time courses for each subject.

### Model definition and evaluation

To build predictive models, we employed two frameworks: a linear model using Pearson's *r* to assess whole-brain FC (Rosenberg et al., 2016; Shen et al., 2017) and a partial least square regression (PLSR) model using accordance and discordance scores to assess whole-brain FC (Meskaldji et al., 2015b, 2016).

### CPM with pearson's *r* as connectivity measure

Pairwise correlations were computed between all pairs of the 268 nodes, and Pearson's *r* correlation coefficients were Fisher *z*-transformed to yield symmetric 268 × 268 connectivity matrices.

To assess the relevance of FC (measured with Pearson's *r*) to behavior, we applied the CPM approach, described in detail in previous work (Finn et al., 2015; Rosenberg et al., 2016; Shen et al., 2017). The following steps were performed in MATLAB (R2016b, MathWorks). First, Spearman's rank correlation between each functional connection, or edge, in the connectivity matrices and ADAS 11 was performed across participants. As suggested by Shen et al. (2017), here we used the Spearman's rank correlation instead of the Pearson's *r* correlation because the distribution of ADAS11 scores in our sample is skewed (Kurtosis = 5.70). The resulting Spearman's *rho* values were statistically thresholded at *p* < 0.01. This edge selection threshold was chosen to remain consistent as in Rosenberg et al. (2016). See Supplementary Figure 1 for the effects of edge selection on the CPM model performance. All the chosen edges were separated into a positive tail (edges whose strength was associated with higher ADAS11 scores) and a negative tail (edges whose strength was associated with lower ADAS11 scores). A single summary statistic, network strength (i.e., the sum of all edges in the positive or negative tail), was used to characterized each participant's degree of connectivity in the selected positive edge set and negative edge set.

To determine whether network strength predicted ADAS11 scores in novel individuals, a leave-one-out cross-validation (LOOCV) procedure was employed. In each set of *n*-1 participants, two linear models were fit relating positive and negative network strength to ADAS11 scores. These models were used to predict the left-out individual's ADAS11 score from the strength of his or her positive and negative network. The Spearman's rank correlations between observed and predicted ADAS11 scores were used to assess predictive power. Non-parametric *p*-values were calculated based on 10,000 permutation tests.

### PLSR with accordance and discordance scores as connectivity measures

In addition to using Pearson's *r* correlation, which computes how correlated the activities of two ROIs are on average, as a measure of functional connectivity, we also calculated two recently developed functional connectivity measures, accordance and discordance (Meskaldji et al., 2015a, 2016). Accordance measures how much two ROIs are co-activated and co-deactivated at the same time, whereas discordance measures how often the activities of two ROIs are decoupled. In this way, accordance and discordance disentangle the correlated and anti-correlated elements in the BOLD activity timecourses of each pair of ROIs.

To calculate accordance and discordance, the mean timeseries of each ROI $\left(z_{i}=z_{1}^{\left(i\right)},\;z_{2}^{\left(i\right)},\ldots \;z_{T}^{\left(i\right)}.\;i=1,\;2,\;\ldots ,\;268\right)$ was normalized by subtracting the median and dividing by the median absolute deviation of each time course as in Meskaldji et al. (2016). To keep only significant activations or deactivations, each ROI time course is compared to a positive threshold (*u*) and negative threshold (*l*) based on the quantile *q* = 0.8 applied in Meskaldji et al. (2016). An activation vector, *z*^*u*^, is constructed such that for all $t\;\in \left\{1,\;\ldots ,\;T\right\}:z_{t}^{u}=0\;if\;z_{t}<u$ and $z_{t}^{u}=1\;$otherwise. Similarly, a deactivation vector *z*^*l*^ is constructed such that for all $t\;\in \left\{1,\;\ldots ,\;T\right\}:z_{t}^{l}=0\;ifz>l$ and $z_{t}^{l}=-1\;$otherwise. The accordance *a*_*i, j*_ and discordance *d*_*i, j*_ values between two ROIs *i, j*, with the corresponding normalized time courses *z*_*i*_ and *z*_*j*_ are given by

(1) $$\begin{array}{r} a_{i,\;j}=\frac{z_{i}^{u}*z_{j}^{u}+\;z_{i}^{l}*z_{j}^{l}}{\sigma_{i}\sigma_{j}} \end{array}$$

(2) $$\begin{array}{r} d_{i,\;j}=\frac{z_{i}^{u}*z_{j}^{l}+\;z_{i}^{l}*z_{j}^{u}}{\sigma_{i}\sigma_{j}} \end{array}$$

where

(3) $$\begin{array}{r} \sigma_{i}=\sqrt{\left(z_{i}^{u}*z_{i}^{u}+\;z_{i}^{l}*z_{i}^{l}\right)} \end{array}$$

For a given timecourse *z***,** the following is true: *a*(*z, z*) = 1, *a*(*z*, −*z*) = 0 and *d*(*z, z*) = 0, *d*(*z*, −*z*) = −1. Note that the discordance score is always negative. The more negative it is, the more often the BOLD timecourses of two ROIs are decoupled. In this way, we obtained a 268 × 268 accordance matrix and a 268 × 268 discordance matrix for each participant.

As in Meskaldji et al. (2016), we then tested whether accordance and discordance predicted ADAS 11 scores using partial least square regression (PLSR). Partial least square regression is particularly helpful for predictive models when the input factors are large in numbers and highly collinear. Briefly, PLSR assumes that there are some lower-dimensional, latent structures behind the factors and thus projects the factors (X) and the response (Y) to a latent space such that the response variation can be explained as much as possible. We reduced dimensionality by selecting only the highest loading component. Increasing the number of PLSR components did not improve prediction performance (see Supplementary Figure 2).

As described in the CPM section above, LOOCV was also employed such that the model was built on *n* − 1 participants and tested on the left-out individual. Spearman's rank correlation between observed and predicted ADAS11 scores were used to assess predictive power. The non-parametric *p*-values were calculated based on 10,000 permutation tests.

### Education-control methods

Because we observed a correlation between years of education and ADAS11 scores in our sample, we ran additional analyses to control for the effect of educational level on prediction.

For CPM, we applied the following two methods separately. First, we included educational level as a control variable during the edge selection of CPM. In this case, the predicted networks consisted of only edges that showed significant partial Spearman's rank correlation with ADAS11 while controlling for years of education. Second, we correlated edge strength with years of education and ADAS 11 using Spearman's rank correlation, respectively. We excluded any edges significantly related to education level (either positively or negatively; *p* < 0.01) from our predictive model of ADAS11.

For PLSR, we controlled for the effect of education by a method similar to the second one mentioned above such that we excluded any edges that showed significant Spearman's rank correlation with years of education in both the accordance and discordance matrices. The resulting matrices were then submitted to PLSR.

### Functional anatomy

We then sought to identify and compare the most important edges selected by CPM and PLSR. We defined final positive and negative CPM networks using data from all participants, restricting each to the 10 edges (out of (268 × 267 ÷ 2 =) 35, 778 total possible edges) most strongly correlated (Spearman's *rho*) with ADAS11 scores in the positive and negative directions, respectively. For the PLSR model with accordance and discordance scores, we obtained the 10 most important edges for accordance and discordance measures separately. For each measure, we first calculated the mean PLSR coefficient across the LOOCV procedure for every edge. We then selected only 10 edges with the most positive mean PLSR coefficients and 10 edges with the most negative mean coefficients. In this way, we obtained three pairs of masks (a positive one and a negative one, with 10 edges each), for the CPM with Pearson's *r* model, the PLSR with accordance model and the PLSR with discordance model.

To explore the functional anatomy of different networks, we summarized the distribution of nodes and edges in two ways. First, we grouped the 268 nodes into anatomically defined macroscale regions (e.g., prefrontal cortex, motor cortex etc.) and then calculated the relative numbers of connections identified by the different predictive models within a region or between each pair of regions. Second, we grouped nodes into eight canonical networks identified by Finn et al. (2015) and examined the relative levels of within- and between-network connections identified by the different predictive models.

## Results

### Predicting ADAS11 with CPM using pearson's *r* as connectivity measure

**Figure 1:**
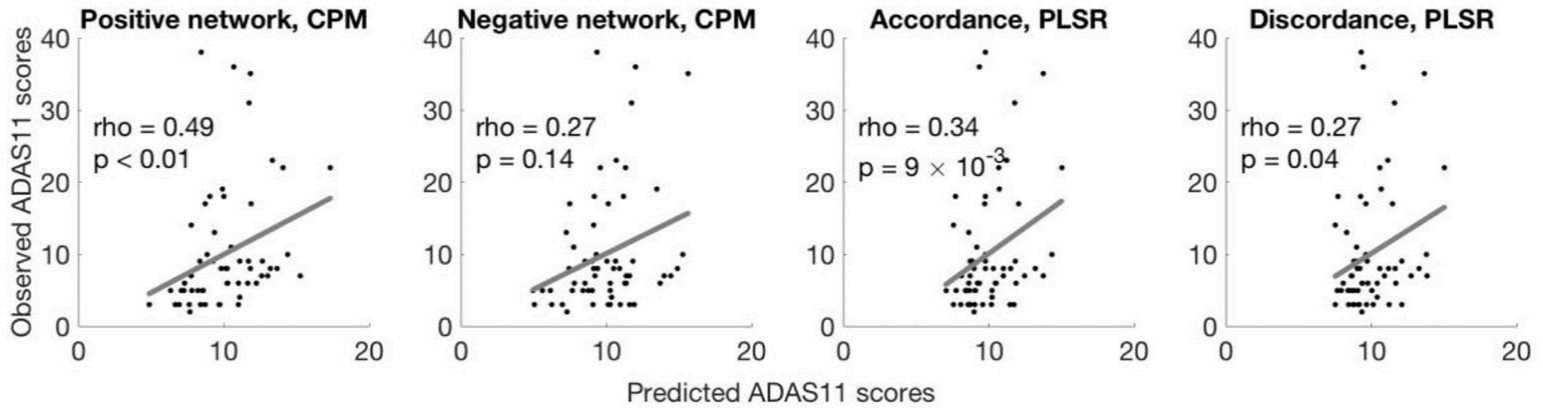
Different models predict ADAS11 scores. Scatterplots show the correlations between the observed ADAS11 scores and the ADAS11 scores predicted by different models for each left-out individual (left to right: positive network defined using CPM, negative network defined using CPM, using accordance in PLSR, using discordance in PLSR). The *p*-values were calculated based on 10,000 permutations.

The CPM based on positive network strength significantly predicted novel participants' ADAS11 scores from their resting-state data (rank correlation between predicted and observed ADAS11 scores, *rho* = 0.49, permutation *p* = 0.009; see Figure 1). However, the negative network model did not yield significant prediction (*rho* = 0.27, permutation *p* = 0.149; see Figure 1).

### Predicting ADAS11 with PLSR using accordance and discordance as connectivity measures

Models built with PLSR separately based on accordance and discordance also significantly predicted ADAS11 scores (rank correlation between predicted and observed ADAS11 scores, accordance: *rho* = 0.34, permutation *p* = 0.009; discordance: *rho* = 0.27, permutation *p* = 0.040; see Figure 1).

### Education control

CPM performance remained similar to the non-education-controlled results after we controlled for years of education using the two different methods described above. After including years of education as a control variable in the edge selection stage, the resulting positive network still significantly predicted novel participants' ADAS11 scores (*rho* = 0.51, permutation *p* = 0.004) while the negative network did not (*rho* = 0.25, permutation *p* = 0.200). In the second method, we excluded any edges that were correlated with years of education from our predictive models. Across all iterations of LOOCV, 1 to 3 (Median = 2) edges were excluded from the positive network and 0 to 2 (Median = 1) edges were excluded from the negative network. With this approach, the correlation between observed and predicted ADAS11 scores was also close to what we obtained above (positive network: *rho* = 0.47, permutation *p* = 0.012; negative network: *rho* = 0.29, permutation *p* = 0.156).

Similarly, after excluding any edges correlated with years of education from the accordance and discordance matrices, models built with PLSR yielded prediction performance comparable to what we observed without controlling for education (accordance: *rho* = 0.34, permutation *p* = 0.010; discordance: *rho* = 0.28, permutation *p* = 0.026).

### Functional anatomy

**Figure 2:**
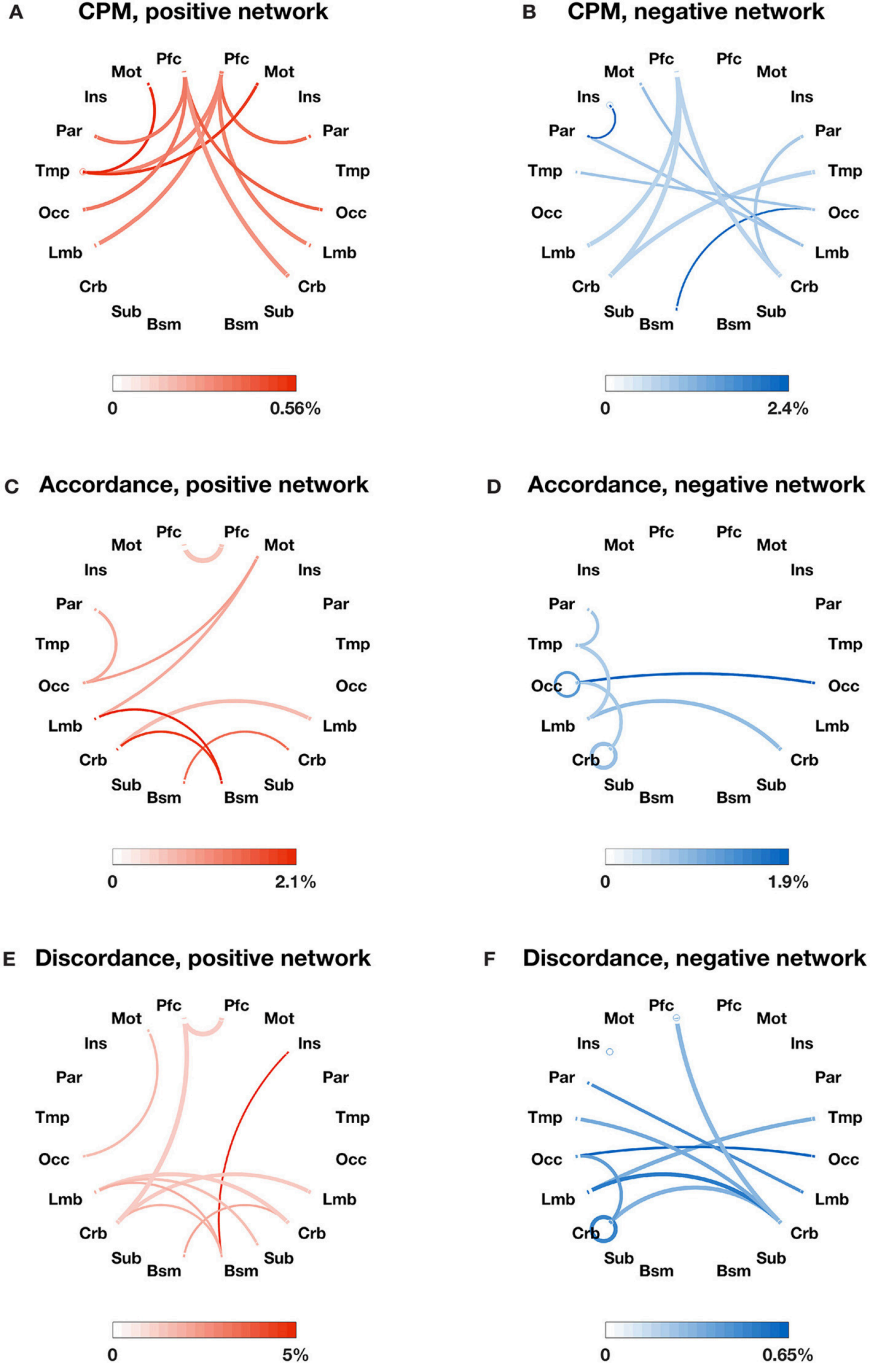
The most important edges selected by different models and their distribution in 10 macroscale brain regions in the left and right lobes. **(A)** Positive edges selected by CPM; **(B)** negative edges selected by CPM; **(C)** accordance edges with positive weights in PLSR; **(D)** accordance edges with negative weights in PLSR; **(E)** discordance edges with positive weights in PLSR; F: discordance edges with negative weights in PLSR. Macroscale regions include prefrontal cortex (PFC), motor cortex (Mot), insula (Ins), parietal (Par), temporal (Tem), occipital (Occ), limbic (including the cingulate cortex, amygdala, and hippocampus; Lim), cerebellum (Cer), subcortical (thalamus and striatum; Sub), and brainstem (Bsm). The circle around each region name represents the total number of possible edges between nodes within the brain region. The line connecting two regions represents the total number of possible edges connecting one node in one region and one in the other. The darkness of the circle/line represents the proportion of edges selected by each model over the corresponding possible edges. The color bar below each plot provides a scale for the darkness of color used in the plot. The percentage below the color bar represents the proportion of edges selected by each model over the corresponding possible edges. Note that discordance scores are negative and a more negative discordance score means the more often the BOLD timeseries of two ROIs are decoupled.

**Figure 3:**
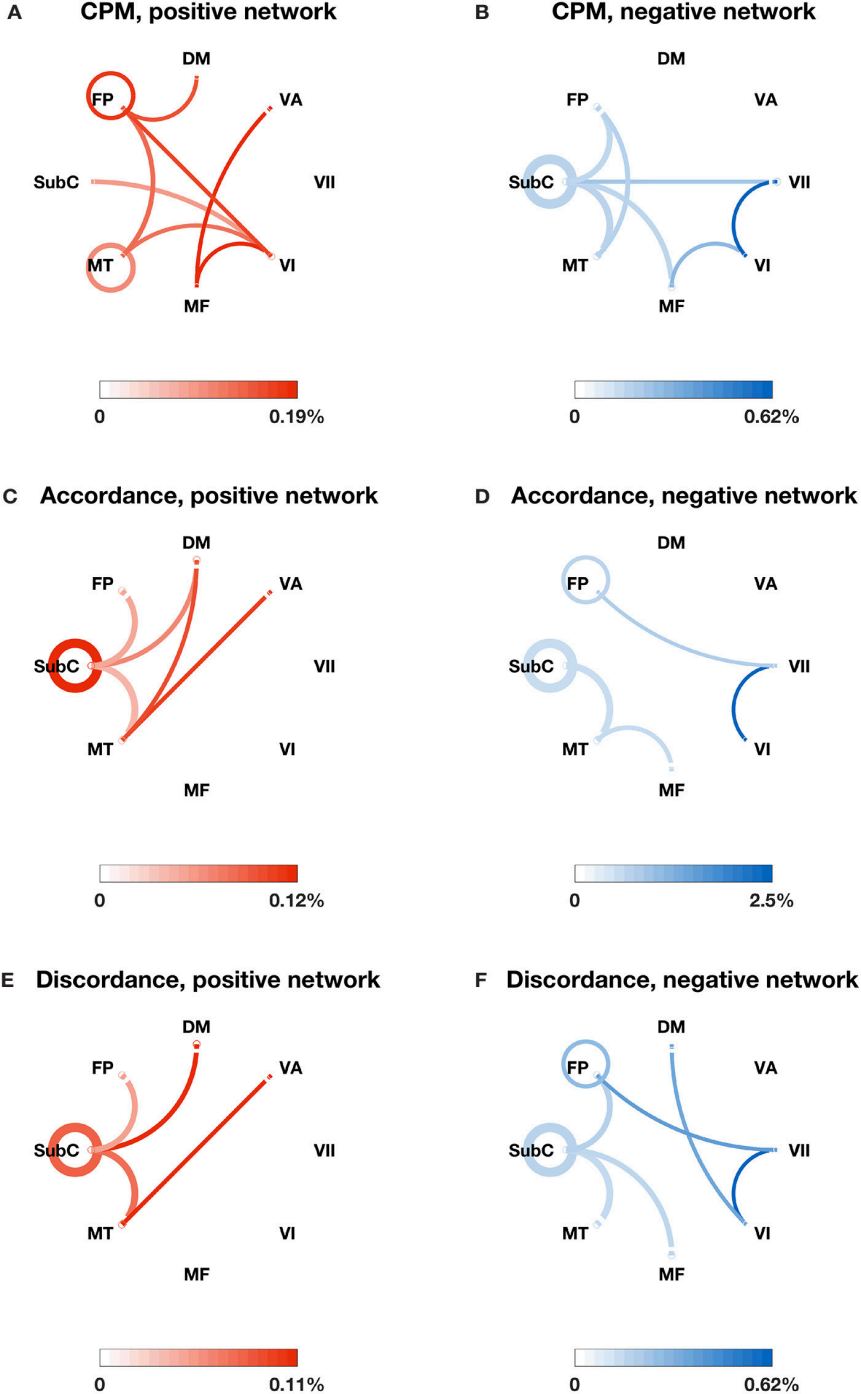
The most important edges selected by different models and their distribution in the eight canonical networks defined by Finn et al. (2015). **(A)** Positive edges selected by CPM; **(B)** negative edges selected by CPM; **(C)** accordance edges with positive weights in PLSR; **(D)** accordance edges with negative weights in PLSR; **(E)** discordance edges with positive weights in PLSR; **(F)** discordance edges with negative weights in PLSR. The canonical networks include the subcortical-cerebellum (SubC), motor (MT), medial frontal (MF), visual I (VI), visual II (VII), visual association (VA), default mode (DM), and frontoparietal (FP). The circle around each network name represents the total number of possible edges between nodes within the network. The line connecting two networks represents the total number of possible edges connecting one node in one network and one in the other. The darkness of the circle/line represents the proportion of edges selected by each model over the corresponding possible edges. The color bar below each plot provides a scale for the darkness of color used in the plot. The percentage below the color bar represents the proportion of edges selected by each model over the corresponding possible edges.

We also identified the most important edges selected by different models (see Figures 2A–F, 3A–F). For the specific distribution of these edges, we focused on the positive network defined by CPM (associated *p*-values for all edges < 4.0 × 10^−4^), which gave the highest prediction performance. Higher strength in this network predicted more severe cognitive impairment. Grouping the 268 nodes into the ten macroscale regions (see Figure 2A) for interpretability, we observed the importance of bilateral prefrontal cortex, left temporal lobe and bilateral motor cortex. Grouping the nodes into the eight canonical networks defined by Finn et al. (2015) (see Figure 3A), we found that important edges fall within the frontoparietal and motor networks. In addition, frontoparietal and visual I networks contained important edges connecting with other networks.

In comparison, the distribution of important edges from the PLSR with accordance model and from the PLSR with discordance model exhibited a different pattern from the CPM positive network, highlighting the importance of brainstem and cerebellum (see Figures 2C–F; associated *p*-values for all edges based on 10,000 permutation tests < 0.005). Similar results were found when the nodes were grouped into the eight canonical networks: the accordance positive network and the discordance positive network both contain edges in the subcortical network, which was not present in the CPM positive network (see Figures 3C,E).

## Discussion

We have demonstrated that resting-state functional connectivity significantly predicts novel individual's cognitive impairment in a highly heterogeneous aging population, ranging from cognitively normal participants to participants with MCI and AD. These promising results suggest that functional networks defined in a data-driven manner contain clinically relevant information about cognitive function and can be developed into markers to capture cognitive decline associated with aging and AD.

We tested different predictive models employing three different measures of functional connectivity (Pearson's *r*, accordance, and discordance) and two different prediction frameworks (CPM and PLSR). The positive network defined by CPM using Pearson's *r* as connectivity measure showed the best numerical performance. However, unlike previous work on fluid intelligence (Finn et al., 2015) and attention (Rosenberg et al., 2016, 2017), in which the positive and negative networks showed comparable levels of predictive power, in our study, the negative network did not predict AD-related cognitive decline. In addition, models built from accordance/discordance measures and PLSR also showed statistically significant prediction performance. Critically, unlike most of previous studies that focus on a priori defined brain regions/networks and compare between groups (Greicius et al., 2004; Sorg et al., 2007; Wang et al., 2007; Bai et al., 2009; Qi et al., 2010; Agosta et al., 2012; Damoiseaux et al., 2012; Vemuri et al., 2012), our analyses were performed in a whole-brain, bottom-up manner. Therefore, our method may particularly useful in identifying features important for predicting cognitive performance on an individual subject level.

In discussing functional anatomy of the edges most relevant to individual differences in cognitive function, we focus on the CPM positive network. A higher strength in this network predicts a higher ADAS11 score and thus more severe cognitive impairment. Consistent with prior work (Wang et al., 2007; Supekar et al., 2008) where rsFC in frontal regions is increased in AD patients relative to controls, we also found that increased FC in prefrontal cortex with other regions is associated with cognitive impairment. We also found that increased rsFC within the frontoparietal network, as well as between the frontoparietal network and default mode network, is associated with worse cognitive performance. This suggests that more impaired subjects may have difficulty alternating between the task-positive and task-negative systems at rest, in line with the recent finding that as AD progresses, there is a reduced anti-correlation between the attentional network and default mode network.

Interestingly, the distribution of important edges identified by the PLSR with accordance model and the PLSR with discordance model is distinct from the CPM positive network and highlights the contribution of subcortical regions to the prediction of ADAS 11 scores. This suggests that the two frameworks may be capturing different aspects of the relationship between rsFC and cognitive decline in aging population. Future work could explore how to combine these two frameworks to build better predictive models.

New work is beginning to show that models based on FC can predict psychiatric diseases before onset. Specifically, FC at 6-months of age predicts the conversion to autism at 24 months of age (Emerson et al., 2017). However, the neuropsychological scale used in the current study, ADAS11, is less sensitive to changes over time and does not show systematic increases related to disease progression (Skinner et al., 2012). If neuropsychological scales more sensitive to temporal changes become available, future work may build models to predict decline in clinically relevant cognitive performance or conversion to AD.

Our study provides promising evidence that functional connectivity from a resting-state scan can reveal AD-related cognitive impairment in an aging population with health, MCI and AD participants, which is potentially advantageous over administering standardized cognitive battery tests that can be challenging and time-consuming. In addition, recent work with other imaging modalities has found promising markers of conversion from MCI to AD (FDG-PET: Pagani et al., 2016; White matter signal abnormality: e.g., Lindemer et al., 2015). Since the ADNI dataset includes multi-modality measurements, future work could explore how to incorporate data from different modalities, such as structural and functional MRI and PET, to establish a comprehensive predictive framework for the cognitive decline in healthy and clinical aging population.

## Ethics statement

Data analyzed here were obtained from the Alzheimer's Disease Neuroimaging Initiative (ADNI) database (Weiner et al., 2010). The ADNI study was approved by the Institutional Review Boards of all of the participating institutions. Informed written consent was obtained from all participants at each site. The current study was carried out in accordance with the Yale University Human Subjects Committee.

## Author contributions

QL, MR, and MC: conceived of and designed the study; QL: preprocessed the data with support and contributions from MR and TO; QL: analyzed the data with support and contributions from MR, KY, and TH; QL: wrote the manuscript with contributions from MR and MC. All other authors commented on the manuscript.

### Conflict of interest statement

The authors declare that the research was conducted in the absence of any commercial or financial relationships that could be construed as a potential conflict of interest.

## Footnotes

^1^ However, other studies with larger samples have found that younger age is associated with lower ADAS-Cog scores (Zec et al., 1992; Doraiswamy et al., 1997b; Peña-Casanova, 1997) and males exhibit modestly lower ADAS-Cog scores (Doraiswamy et al., 1997a).
