# Supplementary material for: Resting-State Functional Connectivity Predicts Cognitive Impairment Related to Alzheimer's Disease
Source: Front Aging Neurosci. 2018 Apr 13;10:94. doi: 10.3389/fnagi.2018.00094 (PMC5908906; doi:10.3389/fnagi.2018.00094)
Supplement: Supplementary file 1 [file DataSheet1.docx]

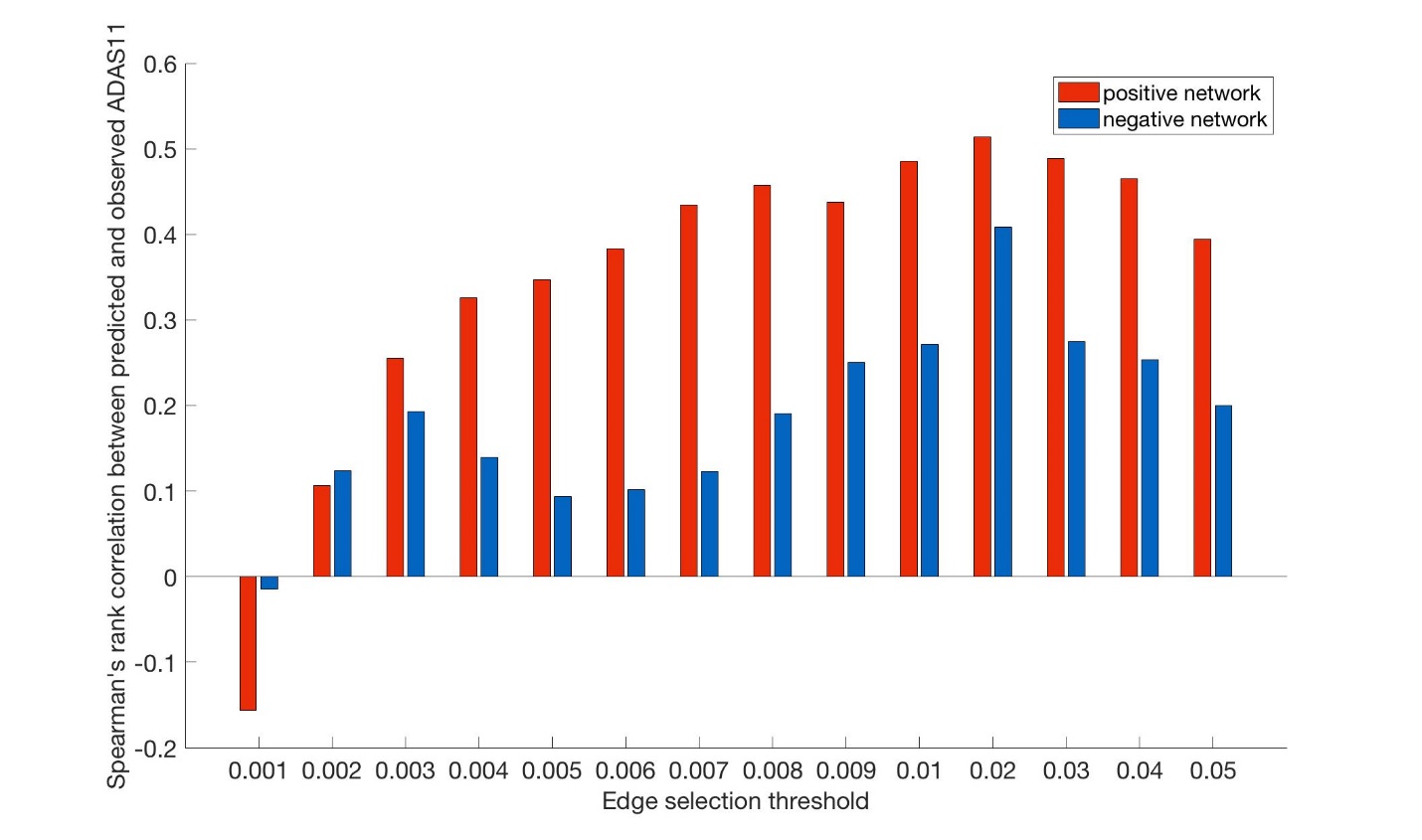


Supplementary Figure 1 The effect of edge selection threshold on CPM performance as measured by Spearman’s rank correlation between predicted and observed ADAS11 scores.


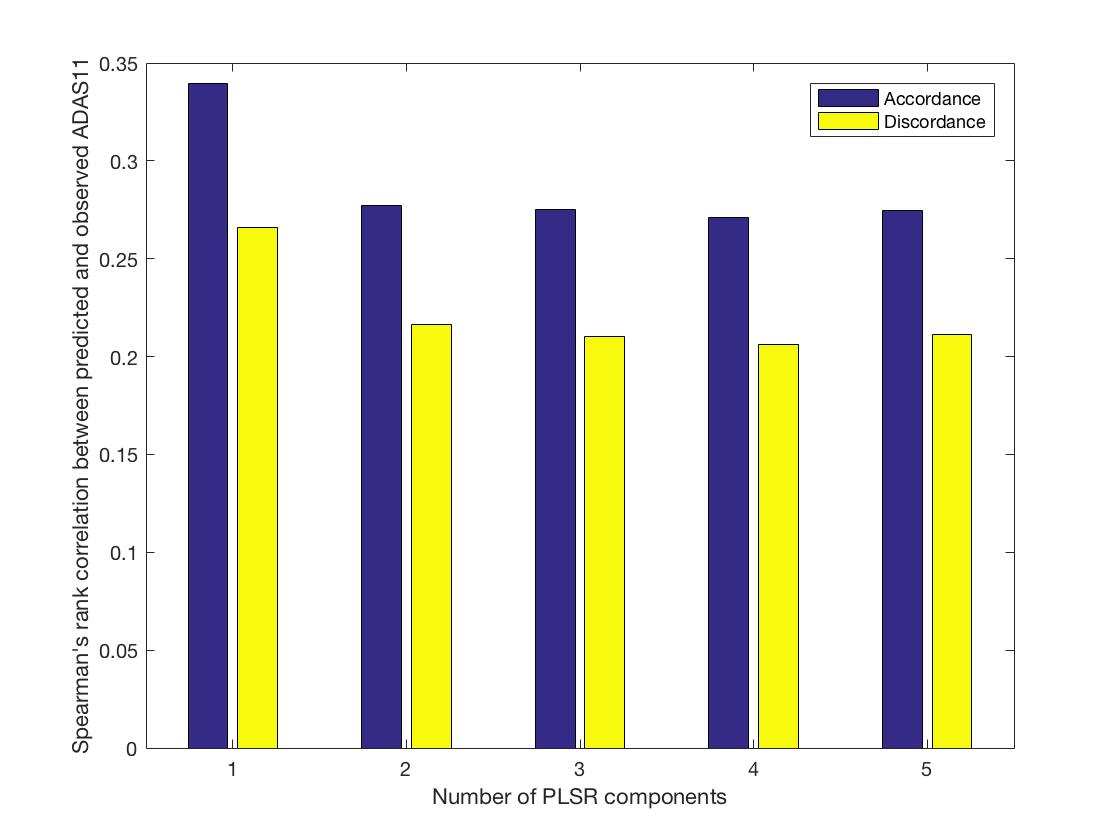


Supplementary Figure 2 Increasing the number of PLSR components did not improve prediction performance as measured by the Spearman’s rank correlation between predicted and observed ADAS11 scores.
